# A Molecular Approach to the Sexing of the Triple Burial at the Upper Paleolithic Site of Dolní Věstonice

**DOI:** 10.1371/journal.pone.0163019

**Published:** 2016-10-05

**Authors:** Alissa Mittnik, Chuan-Chao Wang, Jiří Svoboda, Johannes Krause

**Affiliations:** 1 Max Planck Institute for the Science of Human History, D-07745, Jena, Germany; 2 Institute for Archeological Sciences, University of Tübingen, D-72070, Tübingen, Germany; 3 Department of Genetics, Harvard Medical School, Boston, Massachusetts, 02115, United States of America; 4 Department of Anthropology, Faculty of Science, Masaryk University, Kotlářská, 61137, Brno, Czech Republic; 5 Institute of Archaeology, Academy of Science of the Czech Republic, 69129, Dolní Věstonice, Czech Republic; Universitat Pompeu Fabra, SPAIN

## Abstract

In the past decades ancient DNA research has brought numerous insights to archaeological research where traditional approaches were limited. The determination of sex in human skeletal remains is often challenging for physical anthropologists when dealing with incomplete, juvenile or pathological specimens. Molecular approaches allow sexing on the basis of sex-specific markers or by calculating the ratio of DNA derived from different chromosomes. Here we propose a novel approach that relies on the ratio of X chromosome-derived shotgun sequencing data to the autosomal coverage, thus establishing the probability of an XX or XY karyotype. Applying this approach to the individuals of the Upper Paleolithic triple burial of Dolní Věstonice reveals that all three skeletons, including the individual DV 15, whose sex has long been debated due to a pathological condition, were male.

## Introduction

Sexing of human skeletal material is traditionally performed in archeology by assessing sexually dimorphic traits of the pelvis and skull and, where possible, taking into account typically gendered burial positions and grave-goods. However, the expression of these skeletal traits falls on a continuum and is population-dependent, while gendered burial positions and goods were only common in some cultures and might have reflected different attitudes towards gender and biological sex that we are not aware of. Determination of sex is further complicated if the skeleton is fragmented or incomplete, the individual is sub-adult or when a pathological condition affects the morphology. However, the biological sex of an individual can be assessed by determining the sex chromosomal karyotype. In the case of DNA derived from ancient human remains, a method has been proposed that identifies sex by considering the number of reads in shotgun DNA sequencing data that align to the X and Y chromosomes [1], which is advantageous over previous PCR-based approaches that targeted sex-specific markers [2–4] and that can easily be compromised by modern contamination [5]. However, this method relies on at least 100,000 sequences mapping to the human genome for accurate assignment, a prohibitive requirement for many badly preserved ancient remains. For the majority of prehistoric skeletons a loss of DNA due to post-mortem decay results in a fraction of lower than 0.5% endogenous human DNA in most parts of the skeleton with the rest being mostly comprised of a mix of microbial DNA from bacteria and fungi that settled the body after the individual died [6]. Furthermore, contamination by modern humans, e.g. the excavators or lab technicians, renders the analyses of authentic ancient human DNA difficult.

**Fig 1:**
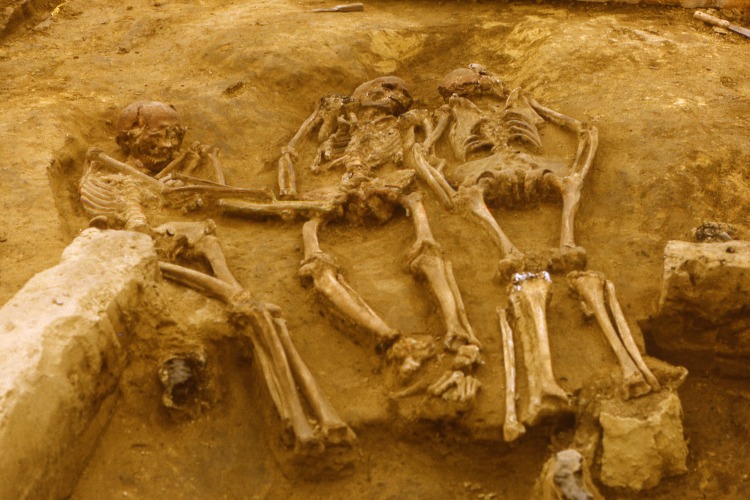
The triple burial of Dolní Věstonice, Moravia, dated to around 31,000 years before present. From left to right: DV 13, DV 15, DV 14.

The Upper Paleolithic triple burial of Dolní Věstonice, site II, (part of the Dolní Věstonice-Pavlov-Milovice site complex in Moravia; Fig 1), dated to 26,640±110 BP (31,155 ± 85 calBP; GrN-14831) [7], has notoriously been difficult to interpret in regard to anthropological sexing. While the two flanking individuals, DV 13 and DV 14, could be identified as a 17–19 and a 16–17 year old male, respectively [8], the individual DV 15 in the middle position of the burial, about 20 years of age, evades osteological sexing due to a pathological, possibly congenital deformation affecting symmetry and proportion of limbs as well as tooth and pelvis morphology [9, 10]. It has been ascribed to both male [11, 12] and female sex [7, 13] and diagnosis of the pathology as the X-linked dominant form of chondrodysplasia calcificans punctata (CCP) has been put forth [14], which is lethal in most cases in males end would thus correspond with a female assignment. A previous study has found a close maternal relationship between DV 14 and DV 15, who carry identical haplotypes for the mitochondrial DNA [15] and kinship between all three individuals has been suggested based on odontological and other non-metric traits [16]. Besides this triple burial and a single male burial DV 16, excavations at Dolní Věstonice II since 1985 have unearthed a structured settlement with a number of stone and bone tools, decorative objects, as well as fragmented remains of associated human individuals making it one of the most important sites of the Central European Gravettian. As extensive rituals seem to have accompanied the burials, the site is especially intriguing for understanding the ideology and social structures of Paleolithic communities [17].

Applying a novel method for genetic sex determination that uses shotgun sequencing data to calculate the ratio of endogenous DNA assigned to autosomes to that assigned to the X chromosomes we are able to establish male sex for all three individuals despite a low fraction of endogenous DNA and the presence of modern contamination.

## Results

Shotgun sequencing resulted in 7,641,368 to 11,902,891 merged and quality filtered reads, of which 2,788 to 16,099 unique reads of 30 bp or longer mapped to the human genome (Table 1). The endogenous DNA content was 0.21%, 0.08% and 0.03% for DV 13, DV 14 and DV 15, respectively. Mapped reads of the samples showed elevated levels of deamination towards the ends (Table 1 and S1 Fig) and a read length distribution shifted toward short reads (S2 Fig), both characteristics of ancient DNA.

**Table 1:** Summary of sequencing results.

Sample	Merged, quality filtered reads	Unique human reads	% endogenous DNA	Cluster factor	% deamination at 5’-end	Contamination on mtDNA^15^
DV 13	7641368	16099	0.21	1.77	27.8	0.9%–2.4%
DV 14	11902891	8945	0.08	1.01	6.4	1.9%–9.2%
DV 15	10298290	2788	0.03	1.83	5.9	0%–3.9%

**Fig 2:**
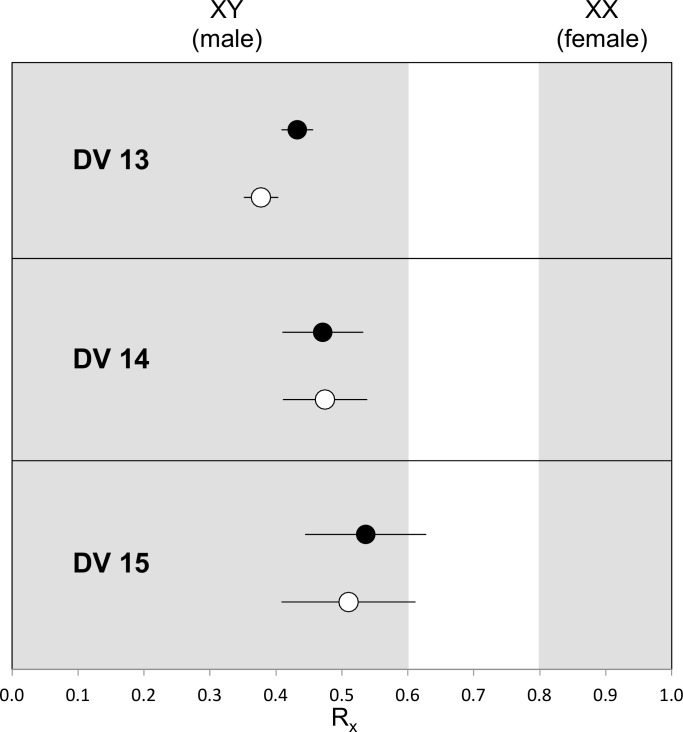
Ratio of alignments to chromosome X compared to ratio of alignments of all autosomes (R_*x*_). An R_*x*_ of below 0.6 indicates male sex, error bars represent the 95% CI. Male assignment is consistent for all three samples for both unfiltered sequences (black dots) and sequences that show evidence of post-mortem deamination (white dots).

We first calculated R_y_ following Skoglund et al.’s method [1] (S1 Table). The chromosomal sexes of DV 13 and DV 14 are consistent with XY but not XX. But R_y_ could not give a sex assignment for DV 15, as there are not enough reads mapped onto the Y chromosome. R_*x*_, defined as the ratio of the alignments to chromosome X to the alignments to autosomes, all normalized against the overall number of alignments to the reference genome, falls below 0.6 for all three individuals indicating male sex (Fig 2, S2 Table). It has been previously established that all three samples exhibit an amount of modern mtDNA contamination that ranges from a maximum of 2.4% in DV 13 to up to 9.2% in DV 14 [15] (Table 1). It can therefore be assumed that modern contamination is also present in the nuclear DNA. After excluding all sequencing reads that did not show any evidence of post-mortem damage in the form of cytosine deamination towards the 5’-end of the molecule, which is a characteristic of authentic ancient DNA [18], filtered reads were consistent with unfiltered reads in giving an R_*x*_ below 0.6 and thus a male assignment for all three individuals (Fig 2).

However, there are only 400 reads left after the above 30bp filtration and an additional post-mortem damage filtration for sample DV15. We caution that the Rx approach is not able to handle well with a sample of only a few hundred reads. To confirm the XY karyotype in DV 15, reads mapping to the Y chromosome were checked. In total 7 sequences securely map to chromosome Y, two of which show cytosine deamination and are thus likely authentic, indicating the presence of ancient Y chromosomal DNA in those remains.

The results obtained here through low coverage shotgun sequencing data are in concordance with the ratio of the number of SNPs mapped on X chromosome and Y chromosome to those mapped on autosomes using genome-wide capture data for those three samples from Dolní Věstonice. They have all been classified as males [19].

## Discussion

While reconstruction and interpretation of pre-historic events is a contentious exercise for archeologists, valuable insights into past social behaviour and attitudes toward death and afterlife can nevertheless be gained through the study of ancient burials.

The triple burial of Dolní Věstonice is especially intriguing due to the peculiarity of the individuals’ positioning–DV 13 on his side facing the central DV 15 with hands reaching the pubic region of the latter, and DV 14 laying face-down. The prominent central position of DV 15 is even more highlighted due to his pathological deformations. The fact that his sex was undeterminable by means of bone morphology and metrics suggests a unique character of this person and such individuals may have received a specific status in egalitarian societies. In addition, the three skeletons were covered partially in ochre and the whole situation was protected by burnt spruce logs and branches, obviously remains of a larger structure.

The sexing of the middle individual DV 15 as male gives us clues to the relationship the dead had to each other. The maternal kinship of DV 14 and DV 15 established by study of the mtDNA [15] as well as their close age raises the possibility of them being brothers. A sibling relationship within the triple burial has been proposed before on the basis of skeletal variants present in all three individuals, however this type of analysis cannot be conclusive, as traits that are rare in extant populations might appear at higher frequency in smaller pre-historic communities due to endogamy [16]. While a close maternal relationship of DV 13 to the other two individuals can be ruled out, a relationship to the degree of paternal half-brother cannot.

The causes of death cannot be established at this point, however, the male assignment of DV 15 rules out some proposed scenarios such as death during childbirth [13]. This individual’s severe pathological condition might have led to his early death. The differential diagnosis as the X-linked form of CCP [14] can now be considered less likely, as male individuals with the condition usually die in early childhood, however, adult male survivors have been reported in the literature and genetic mechanisms for this have been characterized [20, 21].

The molecular sexing of these badly preserved DNA samples was made possible with a novel approach that takes into account the ratio of sequence alignments to chromosome X compared to the autosomes and which gives accurate results with as little as several thousands of reads mapping to the human genome. This method is therefore suitable for light shotgun sequencing data even of samples that contain only a small percentage of endogenous DNA or that are contaminated by modern human DNA. We also see an application of an adapted version of this approach to detect major chromosomal anomalies such as trisomy 21, conditions that were undoubtedly present in pre-historic populations but that can only tentatively be diagnosed in skeletal remains through anthropological methods [22].

## Methods

### DNA extraction and library preparation

All pre-amplification procedures took place in the clean-room facilities at the Max-Planck-Institute for Evolutionary Anthropology in Leipzig, Germany, (DV 13, DV 14) and at the University of Tübingen, Germany, (DV 15) where procedures to minimize contamination with modern DNA are implemented [23, 24]. Sampling on the long bones of the individuals was carried out using a sterile dentistry drill. DNA was extracted from 30–160 mg per sample as previously described [23]. Negative extraction controls were included (S3 Table). From an aliquot of the extract, DNA libraries were made following a modified protocol as described [25]. Each DNA fragment was extended by individual DNA tag combinations corresponding exclusively to one sample to prevent contamination from other sequencing libraries [26]. After amplification of aliquots of these DNA libraries, shotgun sequencing was performed.

### Sequencing and data analysis

High-throughput shotgun sequencing for the libraries of DV 13 and DV 14 was carried out on the Illumina Genome Analyzer IIx platform using 2 x 76 + 7 cycles, for DV 15 on the Illumina MiSeq platform for 2 x 150 + 8 + 8 cycles and for all three libraries on a HiSeq 2500 with RapidRun mode for 2 x 101 + 8 + 8 according to the manufacturer’s instructions for multiplex. Raw sequencing reads were pooled per individual and processed together with a custom pipeline that performs adapter-clipping and merging of reads overlapping at 11 or more bases as well as mapping [27]. The sequences were mapped to the complete human reference genome (hg19/GRCh37/1000Genomes) with BWA 0.6.1 [28].

### Sex assignment

We first used the R_y_ approach [1] to infer the biological sex. R_y_ was performed by computing the number of reads mapped to Y chromosome as a fraction of the total number of alignments to both sex chromosomes.

We here propose a different approach to calculate the averaged normalized ratio of the X chromosome. Let *f*_*1*_, *f*_*2*_,…, *f*_*22*_, and *f*_*x*_ denote the ratio of the alignments to each chromosome to the total number of alignments to autosomes and sex chromosomes, respectively. {*f*_*i*_}(i = 1, 2,…, 22, x) can be estimated directly from the sequenced individual. We then calculated the normalized ratio of each chromosome {*ρ*_*i*_}(i = 1, 2,…, 22, x) by dividing {*f*_*i*_} by the corresponding chromosome ratio of the reference genome used for alignment.

Let *R_x_* be the averaged normalized ratio of X chromosome:

$$R_{x}=\frac{\sum_{i=1}^{22}\frac{\rho_{x}}{\rho_{i}}}{22}$$ (1)

If the shotgun sequencing is completely random, the *R_x_* should be around 0.5 for male samples and 1.0 for females. We tested this approach by assigning several individuals with previous known sex assignments [29–32]. We also follow the approach of Ry [1] to use extreme values as the sex dividing lines. We first performed a linear regression to test if the numbers of sequenced and mapped reads on each chromosome are correlated with the number of reference reads. The assignment result will be invalid if there is no correlation, which means the sequencing is insufficient. We assigned a sample as male if its 95% confidence interval (CI) upper bound for *R*_*x*_ was lower than 0.60 and assigned a sample as female if its *R*_*x*_ 95% CI lower bound was higher than 0.80 (Table 2). The 95% CI was computed as *Rx*±1.96SE. SE is the standard error measuring the amount of variability in the *R*_*x*_ mean compared with 22 autosomes.

**Table 2:** Test of *R*_*x*_ approach for individuals with known sex assignments.

Sample	Nseq	NchrX	NchrY	p-value	*R*_*x*_	95% CI	Assignment
HG00096	129336187	3404137	338394	5.857e-13	0.5313	0.5029–0.5596	XY
HG00099	215786533	10712732	7026	2.200e-16	1.0191	0.9684–1.0699	XX
HG00100	351555180	18015697	12776	2.200e-16	1.0590	1.0024–1.1156	XX
HG00101	185089582	4997300	499215	3.813e-13	0.5523	0.5174–0.5873	XY
Vi33.16	16648258	794453	2813	1.458e-15	0.9551	0.9092–1.0011	XX
Vi33.25	15431136	767200	2124	2.699e-16	1.0042	0.9541–1.0542	XX
Vi33.26	15051507	750642	2098	2.200e-16	1.0109	0.9587–1.0630	XX
Mezmaiskaya-E733	23589975	1114078	3640	1.193e-14	0.9334	0.8864–0.9803	XX
Ajv52	4084279	110151	9607	5.128e-13	0.5478	0.5131–0.5825	XY
Ajv53	861535	44341	119	2.200e-16	1.0636	1.0025–1.1247	XX
Ajv58	95232858	2466677	208840	1.158e-12	0.5159	0.4897–0.5422	XY
Ajv59	214849	5690	505	9.797e-13	0.5306	0.5030–0.5583	XY
Ajv70	7189980	181985	15666	3.529e-12	0.4981	0.4749–0.5214	XY
Gok2	53548001	2429279	2785	1.754e-12	0.8972	0.8382–0.9562	XX
Gok4	1769314	46570	3938	8.424e-13	0.5315	0.4992–0.5638	XY
Gok5	770044	34904	26	6.264e-13	0.9016	0.8460–0.9572	XX
Gok7	562131	24580	26	6.143e-12	0.8685	0.8092–0.9278	XX
Ire8	2274888	60465	4891	7.349e-13	0.5441	0.5060–0.5823	XY
Denisova_4	38626	1021	80	1.491e-12	0.5519	0.5054–0.5984	XY
Denisova_8	828216	21641	1854	1.157e-12	0.5286	0.4939–0.5633	XY

Nseq: number of total alignments; NchrX: number of alignments on X chromosome; NchrY: number of alignments on Y chromosome; p-value: F-statistic p-value in linear regression of the number of reference reads with number of mapped reads.

To further test the minimum number of reads required for *R*_*x*_ sex assignment, we down-sampled reads with a mapping quality higher than 30 from 16 ancient individuals, and found all their sex could be confidently identified down to about 1000 reads (S4 Table).

Patterns of deamination towards read ends were analyzed and plotted with mapDamapge [33]. To account for possible modern DNA contamination the reads were additionally filtered to exclude those that did not show deamination towards the end of the molecule using PMDtools [34] with parameter—threshold 3 (S2 Table). Assignment was also performed with reads with a mapping quality higher than 30.

We provide an R script to compute *R*_*x*_ and assign sex (S1 File).

## Supporting Information

S1 Fig Patterns of deamination towards read ends. (PDF)

S2 Fig Read length distribution. (PDF)

S1 File R script for inference of biological sex using the R_x_ approach. (R)

S1 Table Inference of biological sex using the R_y_ approach. (PDF)

S2 Table Inference of biological sex using the R_x_ approach. (PDF)

S3 Table Summary of sequencing results of negative controls. (PDF)

S4 Table Down-sampled data from ancient individuals to test the minimum amount of reads required for Rx sex identification. (PDF)

S5 Table Sex assignment by artificially adding contaminated reads to the Vi33.26 (female) from a present-day 1000 Genome male sample HG00096. (PDF)
