# Supplementary material for: A Molecular Approach to the Sexing of the Triple Burial at the Upper Paleolithic Site of Dolní Věstonice
Source: PLoS One. 2016 Oct 5;11(10):e0163019. doi: 10.1371/journal.pone.0163019 (PMC5051676; doi:10.1371/journal.pone.0163019)
Supplement: S1 Fig — (PDF) [file pone.0163019.s001.pdf]

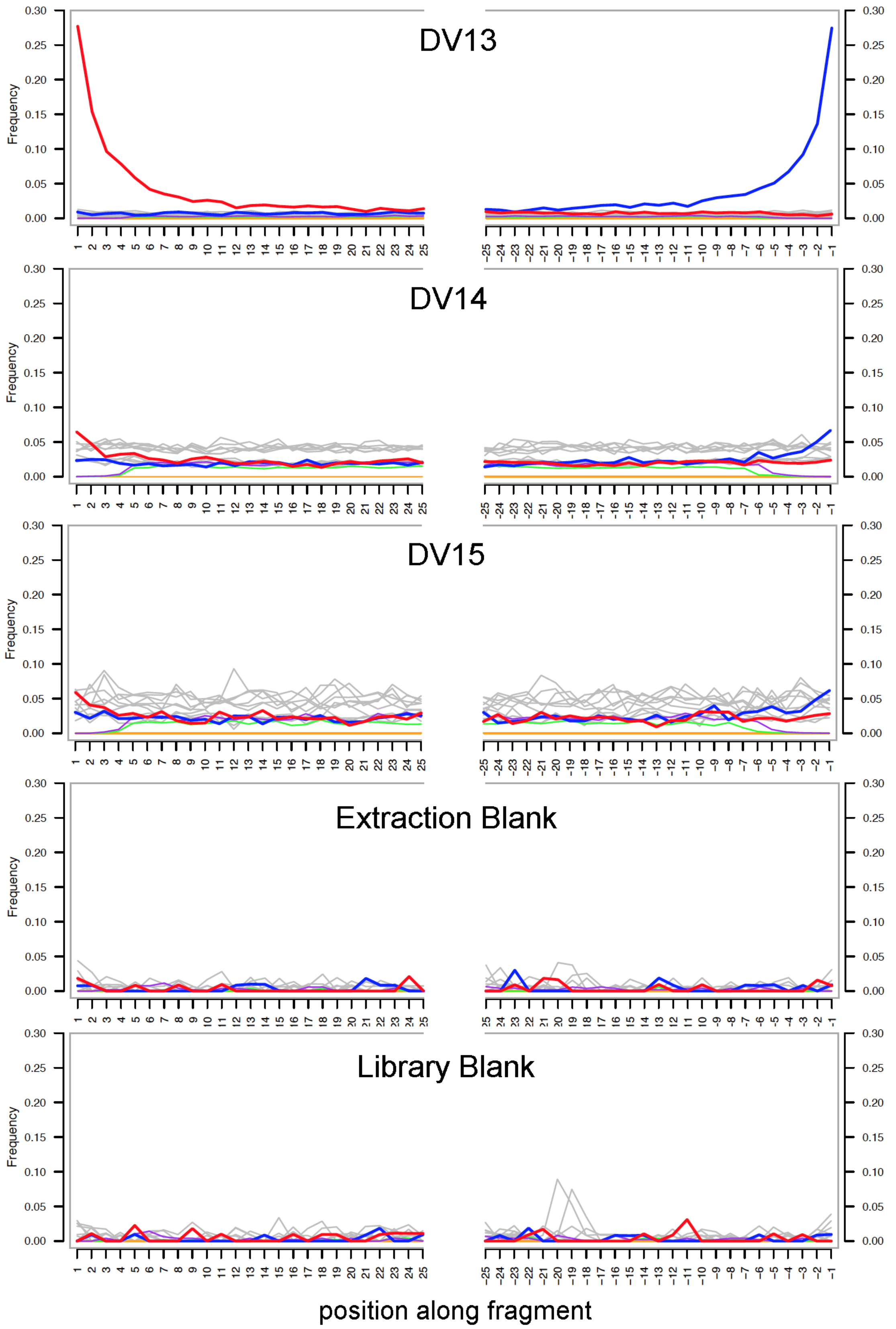


**Figure S1.** Patterns of deamination towards read ends. C-to-T (red) and G-to-A (blue) misincorporations shown at 5'- and 3'-ends, plots generated with mapDamapge.
