# Supplementary material for: A Molecular Approach to the Sexing of the Triple Burial at the Upper Paleolithic Site of Dolní Věstonice
Source: PLoS One. 2016 Oct 5;11(10):e0163019. doi: 10.1371/journal.pone.0163019 (PMC5051676; doi:10.1371/journal.pone.0163019)
Supplement: S2 Fig — (PDF) [file pone.0163019.s002.pdf]

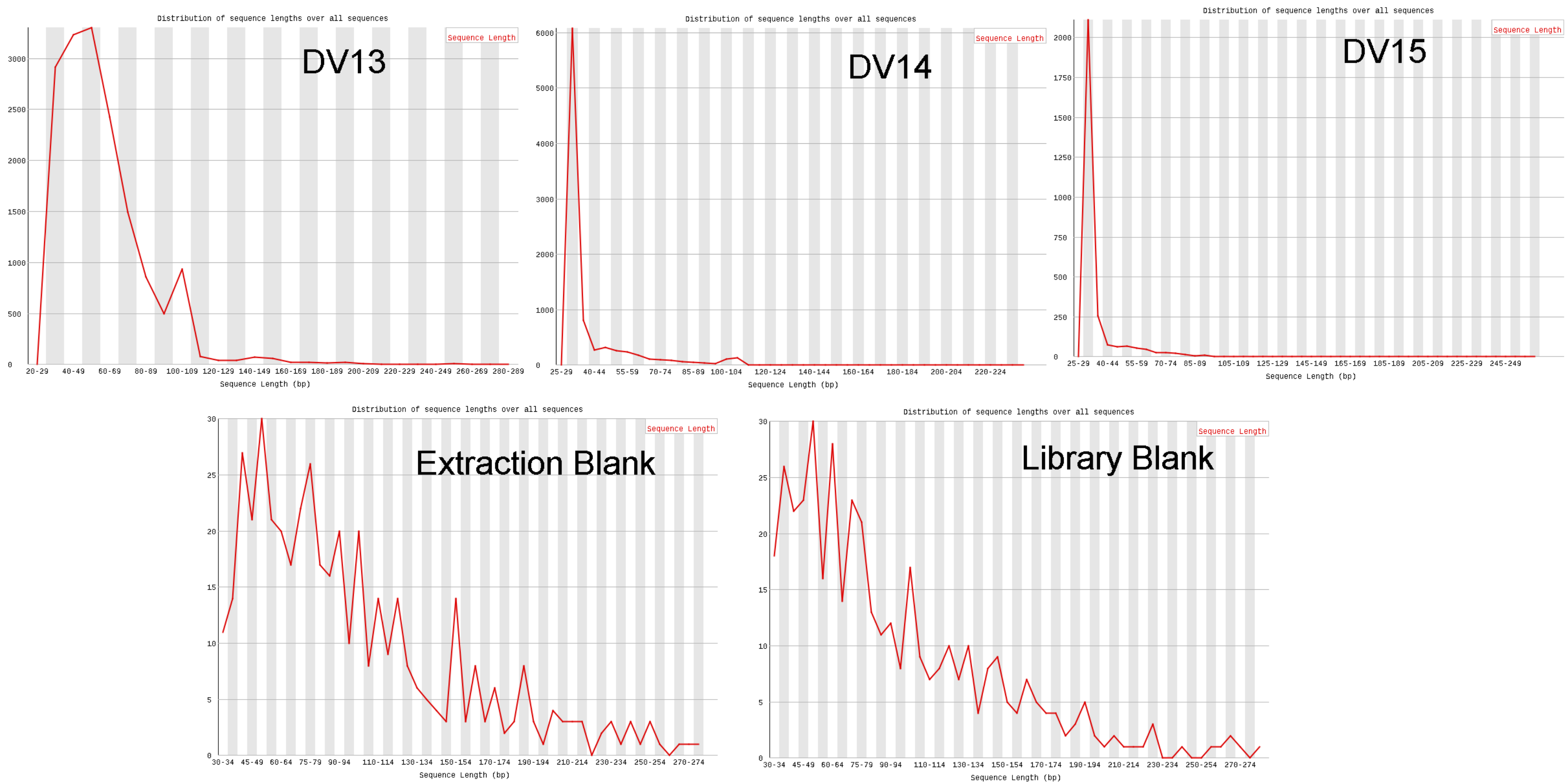

Figure S2. Read length distribution. Distribution of reads mapping to hg19. Negative controls show an excess of unspecific mapping reads under 30bp.
