# Supplementary material for: A Molecular Approach to the Sexing of the Triple Burial at the Upper Paleolithic Site of Dolní Věstonice
Source: PLoS One. 2016 Oct 5;11(10):e0163019. doi: 10.1371/journal.pone.0163019 (PMC5051676; doi:10.1371/journal.pone.0163019)
Supplement: S1 Table — (PDF) [file pone.0163019.s004.pdf]

Table S1. Inference of biological sex using the Ry approach[1]

Sample	Nseqs	NchrY+NchrX	NchrY	R_y	SE	95% CI	Assignment
DV 13	19598	492	42	0.0854	0.0126	0.0607-0.1101	consistent with XY but not XX
DV 14	18527	477	31	0.065	0.0113	0.0429-0.0871	consistent with XY but not XX
DV 15	15034	136	7	0.0515	0.0189	0.0143-0.0886	Not Assigned

Neqs: Number of total alignments; NchrY+NchrX: Number of alignments to chrX or chrY; NchrY: Number of alignments to chrY

References

1 Skoglund, P., Storå, J., Götherström, A. & Jakobsson, M. Accurate sex identification of ancient human remains using DNA shotgun sequencing. *J Archaeol Sci* **40**, 4477-4482, doi:10.1016/j.jas.2013.07.004 (2013).
