# Supplementary material for: A Molecular Approach to the Sexing of the Triple Burial at the Upper Paleolithic Site of Dolní Věstonice
Source: PLoS One. 2016 Oct 5;11(10):e0163019. doi: 10.1371/journal.pone.0163019 (PMC5051676; doi:10.1371/journal.pone.0163019)
Supplement: S2 Table — (PDF) [file pone.0163019.s005.pdf]

Table S2. Inference of biological sex using the R_x approach

>30bp						> 30bp and post-mortem damage filtration				
Sample	mapped reads	mapped to X	mapped to Y	Rx	95% CI	mapped reads	mapped to X	mapped to Y	Rx	95% CI
DV13	16099	368	35	0.433	0.409-0.456	4381	87	11	0.377	0.351-0.404
DV14	8945	215	12	0.471	0.410-0.532	1250	30	1	0.475	0.411-0.538
DV15	2788	73	5	0.536	0.444-0.628	396	9	2	0.510	0.409-0.612
