# Supplementary material for: A Molecular Approach to the Sexing of the Triple Burial at the Upper Paleolithic Site of Dolní Věstonice
Source: PLoS One. 2016 Oct 5;11(10):e0163019. doi: 10.1371/journal.pone.0163019 (PMC5051676; doi:10.1371/journal.pone.0163019)
Supplement: S3 Table — (PDF) [file pone.0163019.s006.pdf]

Table S3. Summary of sequencing results of negative controls

Sample	Merged, quality filtered reads	Unique human reads	% endogenous DNA	Cluster factor	% deamination at 5'-end	Using unfiltered reads			Using reads filtered for post-mortem damage		
						Rx	95% CI	Assignment	Rx	95% CI	Assignment
Extraction Blank	225431	444	0.20	1.97	1.81	1.330	0.778-1.881	consistent with XX but not XY	Too few reads for assignment		
Library Blank	311750	411	0.13	2.17	1.81	0.923	0.829-1.017	XX	Too few reads for assignment		
