# Supplementary material for: A Molecular Approach to the Sexing of the Triple Burial at the Upper Paleolithic Site of Dolní Věstonice
Source: PLoS One. 2016 Oct 5;11(10):e0163019. doi: 10.1371/journal.pone.0163019 (PMC5051676; doi:10.1371/journal.pone.0163019)
Supplement: S4 Table — (PDF) [file pone.0163019.s007.pdf]

Table S4. Down-sampled data from ancient individuals to test the minimum amount of reads required for Rx sex identification

DV13

percentage	Nseq	NchrX	NchrY	p-value	Rx	95% CI	Assignment
1.0%	212	5	1	2.30E-04	0.5972325	0.388166-0.8062991	not assigned
5.0%	1039	29	2	4.22E-07	0.5275569	0.4772645-0.5778494	XY
10%	2061	50	3	1.920E-07	0.460823	0.4197541-0.5018912	XY
20%	3994	100	8	1.492E-08	0.474044	0.4365435-0.5115449	XY
30%	5925	140	11	1.952E-08	0.443472	0.4093505-0.4775936	XY
40%	7891	184	20	2.141E-08	0.438065	0.4046169-0.4715121	XY
50%	9824	220	24	1.419E-08	0.421345	0.3907712-0.4519195	XY
60%	11767	265	28	1.173E-08	0.423889	0.3936594-0.4541184	XY
70%	13774	304	31	1.440E-08	0.415255	0.3856192-0.4448914	XY
80%	15700	364	34	1.232E-08	0.436922	0.4053332-0.4685114	XY
90%	17654	415	37	1.423E-08	0.443202	0.4105451-0.4758591	XY

DV14

percentage	Nseq	NchrX	NchrY	p-value	Rx	95% CI	Assignment
1.0%	194	3	0	3.46E-02	0.3666063	0.2621429 0.4710697	XY
5.0%	930	17	1	1.85E-04	0.3628283	0.3145495-0.4111071	XY
10%	1926	46	3	1.202E-03	0.481507	0.4135979-0.5494159	XY
20%	3832	88	7	1.829E-03	0.46209	0.3949121-0.5292681	XY
30%	5649	123	7	3.347E-03	0.435834	0.3724095-0.4992581	XY
40%	7388	162	8	2.296E-03	0.434617	0.3724277-0.4968059	XY
50%	9242	210	13	1.365E-03	0.450677	0.3864552-0.514898	XY
60%	11075	269	16	1.309E-03	0.481622	0.4138793-0.5493638	XY
70%	13016	313	20	1.504E-03	0.475956	0.4091404-0.5427712	XY
80%	14821	357	24	1.794E-03	0.476904	0.4099107-0.5438973	XY
90%	16614	394	27	1.718E-03	0.468729	0.4034615-0.5339973	XY

DV15

percentage	Nseq	NchrX	NchrY	p-value	Rx	95% CI	Assignment
10%	501	16	2	5.870E-03	0.707859	0.520107-0.8956115	not assigned
20%	1023	26	2	3.560E-03	0.511112	0.4265152-0.5957082	XY
30%	1524	42	2	1.892E-03	0.552494	0.4621639-0.6428245	consistent with XY but not XX
40%	2035	54	3	1.142E-03	0.529557	0.4489556-0.6101582	consistent with XY but not XX
50%	2541	60	4	4.450E-03	0.473994	0.3964223-0.5515666	XY
60%	3074	74	5	7.890E-03	0.483871	0.4050443-0.5626981	XY
70%	3526	84	5	8.992E-03	0.480063	0.4018812-0.5582449	XY
80%	4006	97	5	8.950E-03	0.489678	0.4085511-0.5708057	XY
90%	4521	113	6	8.944E-03	0.507158	0.420028-0.5942882	XY

Vi33.16

percentage	Nseq	NchrX	NchrY	p-value	Rx	95% CI	Assignment
0.005%	831	39	0	8.61E-10	0.996547	0.8714477-1.121646	XX
0.010%	1718	82	0	3.62E-11	0.9903034	0.8936852-1.086921	XX
0.050%	8373	396	0	9.84E-15	0.9505192	0.8971884-1.00385	XX
0.100%	16791	815	1	2.34E-15	0.9731019	0.9250073-1.021197	XX
0.500%	83382	4072	18	1.56E-15	0.9746833	0.9274614-1.021905	XX
1.000%	166186	8004	31	1.83E-15	0.9618736	0.9135125-1.010235	XX
10%	1657146	79410	276	1.362E-15	0.95536	0.9097696-1.00096	XX
20%	3314356	158840	545	1.343E-15	0.95563	0.909802-1.001466	XX
30%	4971989	238512	863	1.403E-15	0.95665	0.9104886-1.002804	XX
40%	6629027	317872	1126	1.419E-15	0.95621	0.9100958-1.002317	XX
50%	8286145	396883	1415	1.430E-15	0.95504	0.908884-1.00119	XX
60%	9944935	476389	1710	1.437E-15	0.95506	0.9089775-1.001151	XX
70%	11604133	555992	1978	1.428E-15	0.95524	0.9092421-1.001247	XX
80%	13265739	635277	2257	1.438E-15	0.95467	0.9087257-1.00062	XX
90%	14920065	714896	2552	1.456E-15	0.95511	0.9091977-1.001021	XX

Vi33.25

percentage	Nseq	NchrX	NchrY	p-value	Rx	95% CI	Assignment
0.005%	773	35	0	1.85E-12	0.9459404	0.8432932-1.048588	XX
0.010%	1496	65	0	6.19E-13	0.8957233	0.8127748-0.9786718	XX
0.050%	7567	387	0	2.04E-15	1.048476	0.974721-1.122231	XX
0.100%	15121	764	2	1.42E-15	1.024428	0.9617273-1.087129	XX
0.500%	76782	3810	11	2.20E-16	0.9995461	0.9477283-1.051364	XX
1.000%	153321	7652	24	2.20E-16	1.005286	0.9539849-1.056586	XX
10%	1535965	76569	210	2.200E-16	1.00347	0.9533489-1.053597	XX
20%	3073797	153292	431	2.250E-16	1.00400	0.953703-1.054302	XX
30%	4610315	229911	630	2.385E-16	1.00373	0.9534349-1.054027	XX
40%	6147146	306620	847	2.478E-16	1.00361	0.9535476-1.053674	XX
50%	7683829	383190	1068	2.546E-16	1.00330	0.9532645-1.053329	XX
60%	9221537	460338	1273	2.579E-16	1.00438	0.954275-1.05449	XX
70%	10757264	537218	1493	2.624E-16	1.00472	0.954645-1.054791	XX
80%	12295263	613929	1698	2.671E-16	1.00442	0.9543832-1.054451	XX
90%	13832268	690630	1879	2.656E-16	1.00444	0.9543176-1.054554	XX

Vi33.26

percentage	Nseq	NchrX	NchrY	p-value	Rx	95% CI	Assignment
0.005%	748	49	0	1.79E-10	1.42903	1.261193-1.596868	XX
0.010%	1532	91	0	2.91E-12	1.253028	1.122092-1.383964	XX
0.050%	7607	395	1	9.11E-16	1.05112	0.998744-1.103497	XX
0.100%	15054	765	2	6.54E-16	1.024862	0.9739389-1.075785	XX
0.500%	75343	3873	9	3.98E-16	1.041435	0.986413-1.096457	XX
1.000%	150888	7688	22	3.97E-16	1.032199	0.9771912-1.087206	XX
10%	1501416	75678	233	2.20E-16	1.02011	0.9667298-1.07349	XX
20%	3002445	150711	435	2.20E-16	1.01503	0.9625238 1.067538	XX
30%	4501553	225852	652	2.20E-16	1.01423	0.9620805-1.066379	XX
40%	6000587	300681	874	2.20E-16	1.01301	0.960837-1.065183	XX

50%	7502505	375559	1079	2.20E-16	1.01184	0.9598288-1.063855	XX
60%	9001408	450541	1282	2.20E-16	1.01176	0.9596738-1.063845	XX
70%	10504031	525848	1463	2.20E-16	1.01189	0.9598176-1.063952	XX
80%	12005484	600665	1678	2.20E-16	1.01133	0.9591612-1.063491	XX
90%	13507881	675714	1894	2.20E-16	1.01118	0.9590279-1.063322	XX

Mezmaiskaya-E733

percentage	Nseq	NchrX	NchrY	p-value	Rx	95% CI	Assignment
0.005%	1160	59	0	2.71E-11	1.020971	0.9378799-1.104062	XX
0.01%	2304	122	0	2.38E-13	1.05654	0.9887535-1.124327	XX
0.05%	11850	583	3	1.09E-13	0.9730139	0.9132497-1.032778	XX
0.10%	23621	1169	5	4.24E-14	0.9779138	0.9258637-1.029964	XX
0.50%	118072	5677	23	1.26E-14	0.9489887	0.901364-0.9966133	XX
1.0%	235646	11197	41	8.87E-15	0.9371548	0.8904988-0.9838108	XX
10%	2350323	111076	361	1.291E-14	0.93098	0.8843402-0.977624	XX
20%	4701873	222403	720	1.249E-14	0.93180	0.8851319 0.9784763	XX
30%	7054119	333845	1092	1.261E-14	0.93248	0.8856109-0.9793581	XX
40%	9407022	446074	1461	1.237E-14	0.93445	0.8875505-0.9813509	XX
50%	11761451	557127	1830	1.232E-14	0.93360	0.8866311-0.9805715	XX
60%	14114485	668501	2212	1.211E-14	0.93352	0.8865398-0.9805043	XX
70%	16469287	779836	2552	1.219E-14	0.93328	0.8862796-0.9802765	XX
80%	18822197	891098	2878	1.209E-14	0.93310	0.8861693-0.9800291	XX
90%	21174747	1002156	3240	1.203E-14	0.93283	0.8858725-0.9797855	XX

Denisova_4

percentage	Nseq	NchrX	NchrY	p-value	Rx	95% CI	Assignment
1.0%	387	18	0	5.01E-10	1.017644	0.9077111-1.127576	XX
10%	3871	146	11	1.198E-13	0.80002	0.7237437-0.8762879	consistent with XX but not XY
20%	7676	240	17	7.419E-13	0.64665	0.5970482-0.6962575	consistent with XY but not XX

30%	11444	348	29	4.998E-13	0.6311	0.579784-0.6824246	consistent with XY but not XX
40%	15371	455	43	7.942E-13	0.61622	0.5669924-0.6654559	consistent with XY but not XX
50%	19307	544	48	9.522E-13	0.58384	0.5382838-0.6293962	consistent with XY but not XX
60%	23177	639	53	1.043E-12	0.57345	0.5257545 0.6211421	consistent with XY but not XX
70%	26947	738	61	1.176E-12	0.57092	0.5220003-0.6198375	consistent with XY but not XX
80%	30772	836	66	1.334E-12	0.56771	0.5173034-0.6181113	consistent with XY but not XX
90%	34598	919	73	2.006E-12	0.55397	0.5054343-0.602513	consistent with XY but not XX

Denisova_8

**percentage**	**Nseq**	**NchrX**	**NchrY**	**p-value**	**Rx**	**95% CI**	**Assignment**
0.10%	844	22	3	1.63E-08	0.537341	0.4814121-0.5932704	XY
0.50%	4134	109	9	1.11E-11	0.534998	0.4942734-0.5757227	XY
1.0%	8327	219	21	6.55E-12	0.532025	0.4951636-0.5688855	XY
10%	82798	2152	189	1.60E-12	0.524	0.4905408-0.5574825	XY
20%	165765	4321	388	1.13E-12	0.526	0.4914798-0.5605984	XY
30%	248185	6516	610	1.13E-12	0.5297	0.4950236-0.56431	XY
40%	330890	8750	791	9.48E-13	0.5338	0.4987424-0.5687977	XY
50%	413459	10903	975	1.16E-12	0.532	0.4972982-0.5667665	XY
60%	496174	13020	1146	1.15E-12	0.5293	0.4946863-0.5640087	XY
70%	578657	15183	1321	1.14E-12	0.5296	0.4946635-0.5644783	XY
80%	661313	17416	1501	1.07E-12	0.5317	0.4964801-0.5669287	XY
90%	743707	19536	1674	1.12E-12	0.5301	0.4952071-0.5650191	XY

Ajv52

**percentage**	**Nseq**	**NchrX**	**NchrY**	**p-value**	**Rx**	**95% CI**	**Assignment**
0.02%	849	24	0	1.31E-12	0.6250158	0.5245244-0.7255073	consistent with XY but not XX
0.05%	1990	47	3	9.46E-12	0.4938456	0.4437614-0.5439298	XY
0.10%	4083	98	5	5.596E-12	0.499709	0.4420859-0.5573321	XY

0.50%	20325	516	54	1.24E-12	0.5101345	0.4782383-0.5420307	XY
1.00%	40310	1063	97	7.665E-13	0.52948	0.4974852-0.5614806	XY
10%	403737	10904	1021	5.039E-13	0.543791	0.5095086-0.5780724	XY
20%	808864	21974	1931	5.228E-13	0.547188	0.5123618-0.5820137	XY
30%	1213600	33168	2892	4.879E-13	0.550079	0.5153011-0.584857	XY
40%	1617609	44068	3857	5.188E-13	0.548413	0.5134458-0.5833793	XY
50%	2023393	55196	4755	4.910E-13	0.549002	0.5141578-0.583846	XY
60%	2427784	66239	5726	4.934E-13	0.548929	0.5142452-0.583613	XY
70%	2832302	77239	6660	5.052E-13	0.54857	0.5139887-0.5831507	XY
80%	3236964	88145	7624	5.111E-13	0.547786	0.513134-0.5824381	XY
90%	3641609	99218	8591	5.121E-13	0.548069	0.513408-0.5827303	XY

Ajv53

percentage	Nseq	NchrX	NchrY	p-value	Rx	95% CI	Assignment
0.10%	825	42	0	2.638E-10	1.036965	0.9553515-1.118578	XX
0.50%	8474	433	0	1.81E-13	1.063543	1.002122-1.124964	XX
1.00%	4245	223	0	4.317E-15	1.03920	0.9818309-1.096574	XX
10%	85077	4459	15	2.504E-16	1.0697	1.007618-1.131779	XX
20%	170561	8930	25	2.200E-16	1.06829	1.006185-1.130394	XX
30%	255434	13353	34	2.273E-16	1.06469	1.004692-1.12469	XX
40%	340897	17930	44	2.200E-16	1.07176	1.010745-1.132777	XX
50%	425687	22333	57	2.200E-16	1.06967	1.008418-1.130931	XX
60%	510138	26721	69	2.200E-16	1.06792	1.007435-1.128411	XX
70%	594717	31005	83	2.200E-16	1.0629	1.002863-1.122938	XX
80%	680005	35478	95	2.200E-16	1.06392	1.003479-1.124355	XX
90%	765666	39914	111	2.200E-16	1.06311	1.002399-1.123827	XX

Ajv70

percentage	Nseq	NchrX	NchrY	p-value	Rx	95% CI	Assignment
0.01%	723	17	2	2.06E-07	0.5265555	0.3979983-0.6551127	consistent with XY but not XX
0.02%	1398	37	2	3.81E-09	0.5703843	0.4560453-0.6847233	consistent with XY but not XX
0.05%	3522	94	8	1.62E-10	0.5345562	0.4780996-0.5910128	XY
0.10%	7088	185	17	2.274E-11	0.5162036	0.4796304-0.5527768	XY
0.50%	35791	879	82	9.52E-12	0.4786992	0.4538688-0.5035296	XY
1.00%	71403	1775	153	7.654E-12	0.4838605	0.4592876-0.5084334	XY
10%	710404	18119	1605	3.573E-12	0.49634	0.4732067-0.5194673	XY
20%	1421733	36307	3191	3.533E-12	0.49676	0.4739195-0.5196086	XY
30%	2133546	54587	4743	3.534E-12	0.4977	0.4747453-0.5206557	XY
40%	2844037	72813	6278	3.324E-12	0.49818	0.4752068-0.5211489	XY
50%	3555449	91065	7859	3.356E-12	0.49843	0.4753636-0.5214891	XY
60%	4267104	109141	9439	3.420E-12	0.49789	0.4746514-0.5211252	XY
70%	4978033	127312	10947	3.507E-12	0.49779	0.4745457-0.5210339	XY
80%	5689336	145528	12516	3.562E-12	0.49778	0.474582-0.5209846	XY
90%	6400159	163772	14115	3.544E-12	0.49800	0.4747118-0.5212897	XY

Gok4

percentage	Nseq	NchrX	NchrY	p-value	Rx	95% CI	Assignment
0.02%	347	7	0	5.53E-09	0.430441	0.378536-0.482346	XY
0.05%	882	15	2	1.03E-10	0.3718528	0.3189595-0.4247461	XY
0.10%	1707	39	4	1.56E-11	0.4596587	0.4265085-0.492809	XY
0.50%	8723	211	25	1.51E-12	0.4858241	0.4534991-0.518149	XY
1.00%	17517	455	55	8.83E-13	0.5225928	0.4895527-0.5556328	XY
10%	175452	4560	414	1.186E-12	0.52064	0.4886705-0.5526025	XY
20%	351756	9251	792	8.677E-13	0.52577	0.4948587-0.5566833	XY
30%	527149	13934	1167	8.051E-13	0.52861	0.4974395-0.5597833	XY
40%	702369	18611	1582	7.681E-13	0.53018	0.4985844-0.5617739	XY
50%	877938	23286	1990	7.846E-13	0.53086	0.4989706-0.5627395	XY

60%	1053125	27936	2362	7.890E-13	0.53091	0.4988983-0.5629283	XY
70%	1228333	32523	2784	8.797E-13	0.52972	0.4976638-0.5617757	XY
80%	1402892	37257	3137	8.433E-13	0.53137	0.4992595-0.5634815	XY
90%	1578923	41967	3547	8.243E-13	0.53202	0.4997071-0.5643345	XY

Gok5

**percentage**	**Nseq**	**NchrX**	**NchrY**	**p-value**	**Rx**	**95% CI**	**Assignment**
0.10%	775	36	0	1.52E-08	0.9678954	0.8332105-1.10258	XX
0.50%	3761	168	1	1.87E-11	0.8757053	0.8151647-0.9362458	XX
1.00%	7511	354	1	1.54E-12	0.9274771	0.8699967-0.9849575	XX
10%	76260	3610	4	7.557E-13	0.93325	0.8742538-0.9922549	XX
20%	152746	7181	6	5.167E-13	0.9259	0.8684543-0.9833475	XX
30%	228642	10720	8	4.342E-13	0.92245	0.8664199-0.9784828	XX
40%	304778	14181	10	4.712E-13	0.91473	0.859332-0.9701317	XX
50%	381087	17654	13	5.015E-13	0.9104	0.8552204-0.9655868	XX
60%	457126	21038	15	5.427E-13	0.90438	0.8493672-0.9593871	XX
70%	532717	24480	18	5.633E-13	0.90337	0.8481203-0.9586204	XX
80%	609003	28017	19	5.660E-13	0.90439	0.8489899-0.9597986	XX
90%	684749	31445	21	6.184E-13	0.90314	0.8472033-0.959085	XX

Gok7

**percentage**	**Nseq**	**NchrX**	**NchrY**	**p-value**	**Rx**	**95% CI**	**Assignment**
0.10%	544	21	0	4.28E-07	0.7703463	0.6853565-0.8553361	consistent with XX but not XY
0.50%	2760	137	0	4.47E-10	0.98671	0.9025912-1.070829	XX
1.00%	5431	264	0	6.27E-11	0.9584038	0.8881479-1.02866	XX
10%	55075	2494	1	8.650E-12	0.8931	0.8288183-0.9573725	XX
20%	110616	4936	1	8.524E-12	0.87814	0.8164303-0.9398433	XX
30%	166300	7418	1	1.005E-11	0.87648	0.8153353-0.9376301	XX
40%	222224	9943	7	9.307E-12	0.88064	0.8185044-0.9427753	XX
50%	277625	12352	8	8.154E-12	0.87498	0.814303-0.9356504	XX

60%	333770	14764	12	7.515E-12	0.86982	0.8099254-0.9297081	XX
70%	389566	17244	14	6.832E-12	0.87056	0.8107012-0.9304268	XX
80%	444747	19614	19	7.058E-12	0.86697	0.8074716-0.92646	XX
90%	501179	22113	23	6.686E-12	0.86741	0.8079989-0.9268158	XX

Ire8

**percentage**	**Nseq**	**NchrX**	**NchrY**	**p-value**	**Rx**	**95% CI**	**Assignment**
0.02%	427	10	0	2.66E-10	0.5435115	0.4315329-0.65549	with XY but not XX
0.05%	1112	26	0	3.95E-12	0.5045376	0.4274472-0.5816281	XY
0.10%	2221	55	1	2.01E-12	0.5202778	0.4496646-0.5908909	XY
0.50%	11403	308	26	1.40E-12	0.5418092	0.5082927-0.5753257	XY
1.00%	22607	607	43	8.71E-13	0.549303	0.5110205-0.5875854	XY
10%	225732	6230	487	4.884E-13	0.56186	0.5220335-0.6016769	consistent with XY but not XX
20%	451304	12279	972	5.334E-13	0.55321	0.5145946-0.591832	XY
30%	677165	18325	1463	6.449E-13	0.55018	0.511359-0.5890057	XY
40%	902223	24273	1960	7.014E-13	0.54639	0.5080203-0.5847599	XY
50%	1127689	30219	2466	7.182E-13	0.54408	0.506302-0.581863	XY
60%	1354133	36158	2934	7.521E-13	0.54218	0.5042427-0.5801267	XY
70%	1579648	42168	3401	7.679E-13	0.54183	0.5040396-0.57963	XY
80%	1805001	48223	3911	7.659E-13	0.54224	0.504543-0.5799337	XY
90%	2031301	54375	4400	7.518E-13	0.54355	0.5055302-0.5815689	XY
