# Supplementary material for: A Molecular Approach to the Sexing of the Triple Burial at the Upper Paleolithic Site of Dolní Věstonice
Source: PLoS One. 2016 Oct 5;11(10):e0163019. doi: 10.1371/journal.pone.0163019 (PMC5051676; doi:10.1371/journal.pone.0163019)
Supplement: S5 Table — (PDF) [file pone.0163019.s008.pdf]

Table S5. Sex assignment by artificially adding contaminated reads to the Vi33.26 (female) from a present-day 1000 Genome male sample HG00096

Percentage	p-value	Rx	95%CI	Assignment
1%	2.20E-16	1.005933	0.9540686-1.057798	XX
5%	2.20E-16	0.986106	0.9352691-1.036943	XX
10%	2.20E-16	0.962039	0.9123184-1.011759	XX
15%	2.52E-16	0.937955	0.8894453-0.9864649	XX
20%	3.03E-16	0.914043	0.8666756-0.9614113	XX
25%	3.89E-16	0.890217	0.8439857-0.9364485	XX
30%	5.22E-16	0.866112	0.8210635-0.9111611	XX
35%	7.43E-16	0.841756	0.7978319-0.8856796	consistent with XX but not XY
40%	1.11E-15	0.817907	0.7751619-0.8606522	consistent with XX but not XY
45%	1.70E-15	0.793689	0.7521696-0.8352082	consistent with XX but not XY
50%	2.64E-15	0.770326	0.7298829-0.8107689	consistent with XX but not XY
55%	4.31E-15	0.74605	0.7068855-0.7852138	not assigned
60%	7.23E-15	0.721947	0.6839785-0.7599163	not assigned
65%	1.22E-14	0.698194	0.6613316-0.7350569	not assigned
70%	2.09E-14	0.674176	0.6385784-0.7097739	not assigned
75%	3.68E-14	0.650088	0.6156972-0.6844791	not assigned
80%	6.40E-14	0.626195	0.5931153-0.6592752	consistent with XY but not XX

Assignment was performed with reads with a mapping quality higher than 30. Percentage: the percentage of random contaminated reads added into Vi33.26; p-value: F-statistic p-value in linear regression of the number of reference reads with number of mapped reads.
